# On Missed Appointments: The Ethics of Nonattendance in General Practice

**DOI:** 10.1111/jep.70222

**Published:** 2025-08-13

**Authors:** Richard C. Armitage

**Affiliations:** ^1^ School of Medicine, Academic Unit of Population and Lifespan Sciences, University of Nottingham, Clinical Sciences Building Nottingham City Hospital Campus Nottingham UK

**Keywords:** ethics, general practice, primary care

## Abstract

**Introduction:**

A substantial number of general practice appointments in England are missed each year, which incurs considerable cost to the NHS. In the absence of an authoritative policy, there is variation in how GPs manage missed appointments in this setting. There are various reasons for why patients miss their GP appointments, many of which lie outside the patients' control.

**Methods:**

This paper undertakes an ethical analysis, using the framework of Principlism, of missed GP appointments in the NHS.

**Findings:**

This paper finds that missed appointments might prevent the patient's autonomy (which requires the health problem for which the GP appointment was booked to be adequately addressed) from being upheld, frustrate the possibility of promoting beneficence (particularly among patients with multimorbidity and mental health problems), threaten non‐maleficence (also particularly among patients with multimorbidity and mental health problems), and violate the principle of justice due to exacerbating health inequalities and wasted scarce healthcare resources.

**Conclusion:**

This paper suggests that GPs should make efforts to contact patients who miss their appointments, via telephone in cases of missed in‐person appointments, and via multiple attempted calls in cases of missed telephone or online appointments.

## 1 Introduction

Since 2018 (when the relevant data became publicly available), between 2.74% and 5.82% of all appointments that take place each month in general practice in England are recorded as Did Not Attend [1]. For example, between December 2023 and November 2024, 16,987,176 (4.3%) of the 396,400,157 total appointments that took place in general practice in England were not attended by the patients they were booked for' [2] (this is the lowest estimation of the true nonattendance rate because the status of 21,310,588 [5.4%] of those total appointments is unknown [2], at least some of which might have been not attended). In 2019, around 7.2 million general practitioner (GP) appointments were missed annually in England, which cost the National Health Service (NHS) over £216 million each year [3].

Various interventions and policies have been introduced that intend to reduce the rate of missed appointments in general practice, including online triage, telephone appointments, and SMS appointment reminders [4, 5, 6, 7]. Some general practices have independently developed their own strategies for managing missed appointments, such as agreeing a limited number of call‐backs for missed telephone appointments and using SMS messaging to communicate with patients [8]. While imposing fees for missed appointments has been considered as a means of reducing the rate of nonattendance [9], a variety of ethical challenges adorn this intervention [10], and it has to date not been introduced in England [11].

Despite the challenges posed by missed appointments in general practice, neither a nationwide policy from the NHS, nor an authoritative guideline from the General Medical Council, has been introduced to standardise and align the management of this phenomenon in England. Accordingly, there is variation in the attitudes towards missed appointments in general practice and in the manner with which they are managed. For example, GPs often consider missed appointments as an opportunity to catch up on time or work [12] in a context of substantial and growing pressures on GPs in the NHS [13]. However, there are various potential medicolegal implications for how missed appointments are managed by GPs [14, 15]. Furthermore, a multitude of ethical issues surround the matter, although this ethical landscape does not appear to have been mapped out to date.

This paper will undertake an ethical analysis of missed appointments in general practice using the framework of Principlism, which comprises of four basic and universal ethical principles—respect for autonomy, beneficence, justice, and non‐maleficence—that are each equally important prima facie moral obligations for doctors in the provision of patient care. The analysis will be restricted to the UK's NHS (the country's publicly funded, single payer healthcare system that is funded by general taxation) and missed appointments with GPs, although the findings might also be applicable to other publicly funded healthcare systems and missed appointments with other health professionals in general practice. The paper will subsequently offer suggestions for practice in light of its findings.

## 2 Ethical Analysis

### 2.1 Respect for Autonomy

Autonomy requires the patient's capacity for self‐determination, and their capacity to make independent decisions in the absence of undue pressure, solicitation or coercion, to be respected [16]. For the autonomy of a patient for whom a GP appointment is booked to be respected, their health concern must be adequately addressed by the GP. A patient might choose to miss their GP appointment because their health condition has resolved or sufficiently improved such that they no longer require the attention of a GP (general practices that offer appointments more than 2 days later than the point at which they are booked typically experience higher rates of missed appointments [17, 18], which might be explained by GP appointments being booked for self‐limiting conditions that resolve or sufficiently improve before the appointment taking place). While such patients exercise their autonomy by not attending GP appointments they no longer wish or need to attend, this practice is ethically suboptimal because it prevents the benefit of the appointment (which is a scarce resource) being availed by another patient.

However, there are various other reasons why GP appointments might be missed by the patients they are booked for. While common reasons for missing appointments, such as forgetting about and difficulty cancelling the appointment [4, 19], might imply that the patient believed they no longer needed to see the GP, this is not necessarily the case (e.g., the patient might have overlooked the appointment due to a busy schedule or the occurrence of an unexpected event). Furthermore, even if the patient did believe the GP appointment was no longer necessary and missed it as a result, the appointment might still be required for their health concern to be adequately addressed by the GP (and, therefore, for their autonomy to be respected), such as if a review of a newly started medication, discussion of a blood test result, or repeat measurement of the patient's blood pressure is required for the patient's care to be clinically effective and safe.

Other reasons for missing appointments clearly lie outside the patient's control and strongly suggest that the patient did wish to attend the appointment but was prevented from doing so, such as an inconvenient appointment time (e.g., the patient was unable to arrange the necessary time off work or childcare to attend the appointment) [20], being too ill to attend [19], transport difficulties, challenging weather conditions, and the appointment not being with the patient's preferred GP [20]. Additional factors might also explain why appointments are missed by those who are unable to attend independently (such as children and adults who require the support of a carer to do so) despite them still needing to consult with the GP. In each of these cases, the autonomy of the patient who misses the appointment has not been sufficiently upheld because their nonattendance has prevented their health concern from being adequately addressed by the GP.

The modality of GP appointment discussed so far has been in‐person appointments during which the patient physically attends the general practice and consults with the GP face‐to‐face. Particularly since the COVID‐19 pandemic, however, the proportion of consultations in general practice that take place via telephone or online has increased substantially [13]. In addition, the recent broad uptake of a ‘total triage’ model in primary care in England usually involves the patient waiting to be called back (via telephone or online, i.e., a remote consultation) by the GP without a fixed appointment time, meaning such patients must often wait and make themselves available over a period of hours to receive the call [8]. While total triage is viewed by general practice staff as a potential means to reduce missed appointments [8], the requirement to often make multiple calls to the patient to achieve this is viewed as a disadvantage [21].

While nonattendance of a pre‐booked, time scheduled, in‐person appointment is simple to discern, it is more difficult to ascertain whether an unanswered telephone call reflects a patient's intention to not engage with the appointment due to their illness having resolved or whether they fail to answer for another reason. For example, a missed telephone appointment might be due to an invalid or no longer utilised telephone number, poor mobile phone signal, landlines being used by other people, and the patient not being near the phone or otherwise being unable to answer it at the time of the appointment due to work commitments or other responsibilities (especially when no fixed appointment time has been prearranged) [8]. As such, what constitutes a missed telephone or online appointment is less clearly distinguished than in‐person appointments that the patient fails to attend.

### 2.2 Beneficence

Beneficence requires doctors to act for the benefit of the patient, such as preventing or removing harm, or the active promotion of some good, such as health [16]. Given that GP appointments are booked for patients to have their health concerns addressed, those appointments constitute opportunities for the GP to promote beneficence by adequately addressing those health concerns and, thereby, actively promoting health. Missed GP appointments can lead to unresolved medical problems, which leave those patients in a vulnerable position and cause them to present to medical professionals at a later point in a deteriorated state of health, or to continue living with an untreated medical problem or with deteriorating health [22]. As such, when patients miss GP appointments for any reason other than their health problem having resolved or sufficiently improved such that they no longer require the attention of a GP, the opportunity to promote beneficence is thwarted, as it can lead to health concerns that are unaddressed, untreated, and unresolved.

Furthermore, it is known that patients who are most likely to miss GP appointments have multimorbidity [18, 20], particularly those with mental health conditions (this is after controlling for the number of appointments made for each patient) [18, 23]. These patients are at significantly greater risk of all‐cause mortality, which increases in a dose‐dependent manner in response to the number of appointments missed. For example, patients with long‐term mental health conditions who miss more than two appointments each year have been shown to have a greater than eightfold increase in risk of all‐cause mortality compared with those who missed no appointments (those who missed appointments died prematurely, and often due to nonnatural external causes, such as suicide) [18]. Accordingly, the magnitude of the missed opportunities to promote beneficence that result from missed GP appointments might be greatest among patients with multimorbidity, and especially among those with mental health conditions, as they are most likely to require GP appointments (to address their multimorbidity), to miss those appointments, and to experience negative health outcomes.

### 2.3 Non‐Maleficence

Non‐maleficence requires doctors, through their medical interventions, to avoid causing harm to their patients [16]. As it is known that missed GP appointments can lead to unresolved medical problems and deteriorating health [22], the GP might have a duty to attempt to contact any patient who misses their appointment, as to not do so might be regarded as to cause harm to the patient through inaction. This perspective is also supported by the General Medical Council's document of expected standards for doctors ‘Good Medical Practice,’ in which it instructs doctors that ‘you must make the care of patients your first concern’ [24]. On this view, responding to a missed appointment with inaction might be considered to fall short of this standard, as the potential for harm to come to the patient as a result of the missed appointment is understood. This violation of non‐maleficence might be greatest among missed appointments by patients with multimorbidity, and particularly in those with mental health problems, because they are both most likely to miss GP appointments and to experience negative health outcomes if they miss them [18].

### 2.4 Justice

The principle of justice requires doctors to ensure that the benefits and costs of actions are fairly distributed between patients [16]. It is known that some groups of patients, such as those with work commitments and childcaring responsibilities, often miss in‐person GP appointments due to these practical challenges [25], while others often miss in‐person appointments due to transport difficulties and challenging weather conditions [20]. For some of these patients, the move towards a total triage model in primary care has increased the accessibility of GP appointments [25], although this shift can also increase health inequalities. For example, the need to have a working telephone, functioning internet connection, and adequate digital competency (20% of the UK population lacks basic digital skills or does not use digital technology at all, and these people are likely to be older, less educated, and in poorer health than the general population) [26] are barriers to attending telephone or online GP appointments for those who do not have access to them [8]. Accordingly, the GP might have a duty to attempt to contact any patient who misses their in‐person appointment, and to offer in‐person rather than telephone or online appointments to those at risk of missing remote appointments, to prevent the deepening of health inequalities and, therefore, to promote justice. It is also known that patients who are most likely to miss GP appointments are young adults [27] and those from low socioeconomic backgrounds [20, 23, 27], while those aged 16‐30 years, those aged over 90 years, and those from areas of high socioeconomic deprivation are more likely to miss multiple GP appointments [28]. Accordingly, the GP responding to a missed appointment by patients from these groups with inaction might violate justice by deepening inequalities in healthcare access amongst these groups.

Furthermore, GP appointments are a scarce resource that, within the context of the UK's NHS (a publicly funded healthcare system), justice requires to be distributed in a fair manner. GP inaction in response to a missed GP appointment (even it if it used to catch up on time or work) [12] leads to this resource being wasted because no patient benefits from it, the total cost of which for the NHS in England exceeded £216 million in 2019 [29]. In this additional way, justice is therefore also violated by missed GP appointments which are responded to by the GP with inaction.

## 3 Suggestions for Practice

This paper has argued that patients for whom GP appointments are booked for health concerns to be addressed must have those health concerns adequately addressed for their autonomy to be respected, and that missed GP appointments threaten this ethical principle and the principles of beneficence, non‐maleficence and justice. Accordingly, the GP might have a duty to undertake particular actions in the event of a missed GP appointment to uphold these ethical principles and avoid their violation. The following actions to achieve this are suggested.

In cases of missed in‐person appointments, the GP should attempt to contact the patient and address their health problem via telephone (e.g., if the patient has not arrived by the time their appointment is due to commence, the GP should call the patient). In cases of missed telephone or online appointments, the GP should attempt to contact the patient via these modalities multiple times, such as three attempts over a 10‐min window during the scheduled consultation time, or three attempts over a 1‐h period during a total triage session. In both cases, a valid telephone number stored in the patient's electronic health record is required, which general practices should regularly maintain, such as each time an appointment for the patient is booked or each time a repeat prescription for the patient is issued. If no valid telephone number is available, the patient should be contacted via letter informing them of the reason that contact was not possible and inviting them to contact the general practice to book another appointment.

## 4 Conclusion

In summary, a substantial number of general practice appointments in England are missed each year, which incurs considerable cost to the NHS. In the absence of an authoritative policy, there is variation in how GPs manage missed appointments in this setting. There are various reasons for why patients miss their GP appointments, many of which lie outside the patients' control. This paper has undertaken an ethical analysis of missed GP appointments in the NHS using the ethical framework of Principlism. It finds that missed appointments might prevent the patient's autonomy (which requires the health problem for which the GP appointment was booked to be adequately addressed) from being upheld, frustrate the possibility of promoting beneficence (particularly among patients with multimorbidity and mental health problems), threaten non‐maleficence (also particularly among patients with multimorbidity and mental health problems), and violate the principle of justice due to exacerbating health inequalities and wasted scarce healthcare resources. This paper suggests that GPs should make efforts to contact patients who miss their appointments, via telephone in cases of missed in‐person appointments, and via multiple attempted calls in cases of missed telephone or online appointments.

## Conflicts of Interest

The author declares no conflicts of interest.

## Data Availability

Data sharing is not applicable to this article, as no new data were collected in this study.
